# Entrepreneurship innovation using social robots in tourism: a social listening study

**DOI:** 10.1007/s11846-023-00646-9

**Published:** 2023-03-25

**Authors:** Luis J. Callarisa-Fiol, Miguel Ángel Moliner-Tena, Rosa Rodríguez-Artola, Javier Sánchez-García

**Affiliations:** Business Administration and Marketing Department, Universitat Jaume I, 12071 Castellón, Spain

**Keywords:** Social robots, Social listening, Sentiment analysis, Tourism, Hospitality, Entrepreneurial innovation, M310 Marketing

## Abstract

The tourism sector has been one of the most impacted by the COVID-19 pandemic, due to restrictions on mobility and fear of social contact. In this context, business innovation through digital transformation is presented as a great opportunity for the tourism industry and the inclusion of social robots in service tasks is an example. This transformation requires new methodologies, skills and talent that must be promoted to improve the innovative tourism ecosystem. With this research, we try to determine how the inclusion of social or service robots in hotels can improve the image and perception held by clients or guests. For that, we first analyse the degree of knowledge and sentiment generated by social robots through a social listening study in social networks. In addition, we determine whether these perceptions on the subject are in tune with other more formal fields, such as scientific research, or with the strategies followed at a national or international level by companies, agencies and organisations related to the technology and innovation of social robotics. For both objectives, we use the Simbiu social listening tool, a software-based program on Talkwalker, and we obtain interesting results. Basically, people on Twitter have a neutral or positive feeling about the use of social robots, and people who write in English have a more positive attitude towards social robots than Spanish speakers. After COVID-19, are necessary changes in strategic decisions of the hospitality and it is essential to continue investigating the role of social robots in this new context.

## Introduction

The tourism sector is one of the most important service sectors in the world. Over the years, it has been characterised by significant dynamism, although this has not prevented it from being one of the most affected by the COVID-19 crisis. (Breier et al. 2021; Crespi-Cladera et al. 2021; Villacé-Molinero et al. 2021; Hidalgo et al. 2022). The consolidation of mature tourist destinations (France with more than 90 million visitors, Spain with 83.3 million, followed by the USA with 79.3 million, and Italy with 64.5 million in respectively) has joined other new destinations with significant growth figures, such as Myanmar (40.2%), Puerto Rico (31.2%), Iran (27.9%), Uzbekistan (27.3%), Montenegro (21.4%) and Egypt (21.1%). According to the "OECD Tourism Trends and Policies 2020", the tourism sector accounts for an average of 4.4% of GDP, 6.9% of employment and 21.5% of service exports in the OECD. However, these figures have changed substantially in the last two years (a drop of 73% in 2020 and a drop of 72% in 2021 compared to 2019, which means that a drop-in income from international tourist arrivals has caused a decrease in export revenues from international tourism between − US$ 0.9 and − US$ 1.0 trillion (UNWTO 2022). The tourism contribution to GDP was estimated at US$1.9 trillion in 2021, which is better than US$1.6 trillion in 2020 but worse than US$3.5 trillion in 2019.

The tourism sector has important challenges. The first is adaptation to the new situation, almost in the next years, where new security protocols will be based on greater health restrictions, increased hygiene and cleanliness of the facilities along with guaranteeing adequate social distance, which may lead to changes in tourist behaviour (Bowen and Whalen 2017; Lu et al. 2021). Some authors, such as Pillai et al. (2021), have called this entire new environment Hospitality 5.0.

The use of robots in tourism is a possible solution to the sanitary protocols of hotels, but their use is still testimonial (Ivanov and Webster 2017a, 2017b, 2020; Hou et al. 2021; Lu et al. 2021; Christou et al. 2020; Seyitoğlu and Ivanov 2020). Robots and artificial intelligence have been used within the category of service robots, public relations robots such as telepresence robots or robots for mobile guidance and information (Meléndez-Fernández et al. 2017). In this regard, for example, robots have developed various functions, such as chefs, receptionists, information points or luggage carriers. (International Federation of Robotics 2022).

Robotic assistance devices (also known as RADs), or service robots sometimes with humanoid aspects, are currently being tested in travel agencies (for example, Pepper from AMADEUS), hotels (for example, Mario from Marriott or Connie from Hilton) and airports (Spencer from KLM or Leo from SITA). Their functionality ranges from interacting with and entertaining travellers to physically assisting them (for example, carrying and checking luggage or directing them to boarding gates).

However, these examples are still a mere anecdote regarding the level and application of robots in the tourism sector. In fact, the little scientific literature on the adoption of this type of technology in these industries (Belanche et al. 2020; Choi et al. 2020; Flavian et al. 2019) highlights that the incorporation and use of robots can mean a revolution in the travel and tourism industry (Ivanov and Webster 2017a), affecting both the creation or disappearance of jobs, as well as the development of operations and the perception of quality of service, which will bring about more or less significant changes both in destination management and in the industry in general (Kraus et al. 2019).

However, beyond the advancement of technology, a key aspect in the adoption of social robots in tourism is their perception by customers (Christou et al. 2020; International Federation of Robotics 2022). In this sense, a key research question involves the feelings and emotions social robots generate among tourists. Social listening and sentiment analysis are among the most interesting research methods used in social sciences and management in recent years. The increased use of social media has enabled companies to have insight into customer thoughts and feelings in order to improve their experiences (Hadjielias et al. 2022; WTTC 2022).

Small and medium-sized companies and entrepreneurs can trust social networks to improve the knowledge of their companies (Khanin et al. 2022; Kraus et al. 2012). Entrepreneurs have limitations in using social networks, either due to technical ignorance about utilising those tools to promote their services and products or a lack of economic resources to hire companies to help them in this task (Nowinski and Rialp 2016; Sharma et al. 2022). They often miss out on the most powerful advantage of social media, that is, social listening. Social does not apply only to large companies. It can also help start-ups and SMEs grow since it is a new way of knowing what you think the market and society think of the products of a brand or company or on the brand and the company in general (Kraus et al. 2023; Sharma et al. 2022).

The importance of the tourism sector in the world economy is beyond doubt, and for some countries, it is a critical source of income for the stability of their economy and social welfare (Crespi-Cladera et al. 2021; Khanin et al. 2022; Villacé-Molinero et al. 2021). Its constant dynamism is a sign indicating a permanent need to innovate and adapt to the changes in its environment, in the economic, social and environmental spheres. The significant increase in the digitalisation worldwide, especially in the most developed countries, has had a special reflection on the tourism sector since it has been one of the most evident changes in the behaviour of tourists or travellers (Filipiak et al. 2020; Hadjielias et al. 2022; Kraus et al. 2022; WTTC 2022).

After all these considerations, the objective of this paper is to analyse the degree of knowledge and sentiment generated by social robots through a social listening study. More specifically, we analyse the opinions, attitudes and behaviours of travellers and tourists in their interrelation with social robots, with the aim of trying to better understand their way of thinking and acting, thereby improving the level of implementation and determining whether these perceptions are in line with other more formal fields. For this, we have based ourselves on the use of a tool such as Simbiu, a sentiment analysis software based on the Talkwalker platform, which has allowed us to analyse three of the most used social networks: Twitter, Facebook and Instagram (Keramatfar and Amirkhani 2019; Stewart et al. 2017).

The structure that we have followed in this article is, first, to carry out an analysis of the existing bibliography on the use of social or service robots in the tourism field. Second, the social Listening and Sentiment Analysis is analysed, including its applications and results obtained in other investigations. Subsequently, in point 3, the methodology followed for sentiment analysis with Simbiu software and how the data were collected are explained. Finally, in point 4, the results are collected, and in point 5, the main discussion and conclusions of this study are presented.

## Theoretical framework

### Social robots in the touristic context

The success of a tourism firm depends on several entrepreneurial factors and activities, among which is its innovative capacity to be able to deal effectively and attractively with the new changes and challenges that appear in the sector (La Peña et al. 2016; Mende et al. 2019). In this new scene, new human and non-human actors, such as robots, appear, and consequently, it is very important to analyse interactions between human and nonhuman actors (Glaser 2017; Dannar-Schöerer 2021; Gao and Akbaritabar 2022). In addition to between actors, it will be necessary to continue innovating in aspects such as the development of robotics, automation and artificial intelligence (AI) in the hotel and service industry, making it possible to promote the improvement of this sector (Schneider 2018; Choi et al. 2020; Hou et al. 2021). It is very important to follow research on the relation between humans and non-human actors, such as robots.

The use of social robots in the tourism sector and especially in the hospitality sector has become an important entrepreneurial initiative, offering some services to customers and improving the experiences of tourists and guests (de Kervenoael et al. 2019; Ivanov 2020; Lee et al. 2021). This entrepreneurial initiative could be located within the theory of "work specialisation" identified by Ferreira et al. (2019) in their bibliographical research as one of the six underlying theories of entrepreneurship. This theory is defended by Holmes and Schmitz (1990), who point out that entrepreneurs are people who respond to the needs and requirements of the market and that their ability to capture opportunities derives from being specialists in their tasks.

Social or service robots are defined as fully or partially automated technologies that cocreate value with humans through their social functionalities (Wirtz et al. 2018; Caic et al. 2019). Some of these functions include accompanying hotel guests to their room or to the bathroom, calling a taxi or making a reservation at a restaurant, and informing them about the services offered by the hotel (Belanche et al. 2020). The robot might also offer additional assistance to the customer (Nakanishi et al. 2020). This is especially relevant in the tourism sector, where they perform customer service tasks (Lu et al. 2021; van Doorn et al. 2017; Wu and Cheng 2018).

One of the great challenges of the tourism sector in recent years is the management of tourist experiences where technological innovations, such as the development of AI, the use of big data and the use of social robots, along with a commitment to creating value in the organisation (Amström et al. 2022; Rubio-Andrés et al. 2022), can help generate unique and different experiences (de Kervenoael et al. 2019; Tung and Au 2018). The use of this technology may help companies gain insight into customer needs, which allows us to reach conclusions and establish behaviour patterns based on the traceability of visitors and the link with their social networks and communication channels, among other areas (Flavian et al. 2019; Huang and Rust 2021; LaTour and Brant 2021). These aspects are collected in the MSI Research Priorities 2020–2022.

The acceptance of social robots by users of a service has been approached from different perspectives. It has been approached from the framework of the traditional Technology Acceptance Models (TAM) in which it is established that the perceived ease of use and the perceived usefulness of the technology are basic aspects for its adoption. Its acceptance by employees has also been analysed based on the theory of planned behaviour (TCP), indicating that human behaviour is voluntary and is determined by behavioural intention, which is the result of three main processes: social attitudes, subjective norms and perceived behavioural control (Ajzen 1985: Piçarra 2016). Likewise, it has been approached through the Negative Attitude towards Robots Scale (NARS), which measures the attitudes of humans towards robots (Nomura et al. 2006; Bartneck et al. 2009a; 2009b; Syrdal et al. 2009). Additionally, the Robotic Social Attributes Scale (RoSAS) (Carpinella et al. 2017) aims to help to better understand participants' perceptions of HRI relationships.

The existence of social capabilities (smile, talk, ask, move the eyes, etc.) in robots improves HRI since it generates more emotional engagement and a more fluid interaction with humans (Ivanov and Webster 2020; Tulli et al. 2019). However, its existence by itself is not enough to justify its presence in a service environment. It is important that the social robot is able to provide some kind of clear benefits to companies and that it can also adapt to diverse and unstructured environments (Bartneck et al. 2019), which could improve the expectations for its presence for long periods (Baraka et al. 2020; Leite et al. 2013). Hence, it is important to carry out fieldwork that allows generating greater interaction between consumers, companies and researchers, as is the circumstance in this case (Tulli et al. 2019).

### Social listening and sentiment analysis

Social listening is an active process of attending, observing, interpreting, and responding to a variety of stimuli through mediated, electronic and social channels (Stewart and Arnold 2018). Social listening arises as a result of the rise of online social media and the increase in applications and software capable of analysing what happens in that media. The improvement in this aspect has made it possible to improve the results of capturing and analysing the content generated by users on different digital platforms, both in the professional and social spheres (Ballestar et al. 2020).

This technological improvement has also allowed us to improve the way we communicate and listen to others, which can cause changes both in our digital behaviour and in the physical environment and, therefore, can influence our interpersonal commitment and engagement with brands (La Rose and Brand 2021; Scholz et al. 2021; Stewart and Arnold 2018). This greater communicational intensity as a consequence of the increase in connectivity and interrelation in social networks and an increase in mobile technologies can cause changes in the way we attend to stimuli; for example, it may impact how and where we listen to, when and how we respond to messages and what repercussions it has on third parties who read or listen to them (Ansary and Nik Hashim 2018; Olson and Ro 2020).

The presence of social networks, the generation of content and the development and availability of mobile technologies contribute significantly to the construction of social listening, which becomes more recurrent in an increasingly media and hyperconnected society. Therefore, social listening has clear implications for the behaviour of organisations both among themselves (supplier-manufacturer-distributor/retailer) and in their relationship with customers or final consumers. It can also have repercussions on interpersonal relationships and, thus, can be a very important tool for improving company results, achieving a higher level of customer satisfaction and improving the customer experience (LaTour and Brand 2021).

Social listening is based on the combination of the use of social network monitoring tools with the analysis technology of those networks. The former is generally used to collect, group and classify information from multiple accounts and channels, while social listening analysis tools integrate sentiment analysis with influencer analysis and trend analysis, allowing for relevant information to be obtained from social monitoring in a faster and more effective way.

Consequently, sentiment analysis as a subset of social listening is one of the emerging topics in recent years in the field of marketing. As a result of the appearance of increasingly advanced applications and software that allow analysing and taking advantage of a large amount of content generated by the user, it tries to understand what their feelings, attitudes and behaviours are towards a brand or a brand's products (Saad and Saberi 2017; LaTour and Brand 2021). Notably, social listening and social monitoring are not the same. With social monitoring, the company observes, is aware of, and stays on top of online conversations about itself and its brands primarily to focus on measuring the success of its marketing campaigns and brand reputation management. Social listening involves participating in these conversations in a way that helps to design your online marketing strategy based on the knowledge gained. In other words, social monitoring is the basis for social listening. On the one hand, sentiment analysis analyses mentions and classifies them into positive, negative and neutral emotions. On the other hand, sentiment analysis tries to interpret, delves into the meaning of mentions and classifies them into positive, negative and neutral emotions.

In the literature review, we can find different definitions of sentiment analysis. For example, to Devika et al. (2016), sentiment analysis is the process of collecting or retrieving information about a consumer's perception of a product, service or brand. Saad and Saberi (2017) say that opinion mining (OM) or sentiment analysis (SA) can be defined as the task of detecting, extracting and classifying opinions on something. In the same sense, Medhat et al. (2014) and Mejia and Kajikawa (2017) highlight that sentiment analysis is the task of automatically coding text entries as positive or negative and sometimes neutral. Ligthart et al. (2021) and Liu (2015) describe the computational study of people's opinions, sentiments, emotions, and attitudes towards entities such as products, services, issues, events, topics, and their attributes. In summary, sentiment analysis tries to identify and classify sentiments in the digital world in social media to understand the knowledge of companies and to make better decisions to improve the real experiences of their clients or customers with them and, consequently, improve their economic and reputational results.

In a digital world, the internet is more than a channel for searching for information and making purchases. It is a platform where people generate content expressing opinions, reviews and sharing experiences about products, services, trademarks or companies. In a dynamic and uncertain, confusing and ambiguous world, companies need to know exactly how people feel or think about their business (Muñoz-Pascual et al. 2021; Trischler and Li-Ying 2022). Sentiment analysis might be the best solution (Fang et al. 2018, Devika 2016). For better understanding, sentiment analysis of social media collects the conversations, comments and opinions of customers in the online environment and contextualises them (Appel et al. 2016) with the objective of creating more successful experiences in the relationship with the company (LaTour and Brand 2021).

Sentiment analysis does not subscribe to any specific area of research. Rather, it is an interdisciplinary field that connects natural language processing (NLP), computational linguistics and text mining (Appel et al. 2016). The great challenge of working in sentiment analysis is obtaining reliable information about the opinion, feelings and subjectivity that appears in the texts analysed with the corresponding software.

Among the primary reasons that have led to this growing interest in sentiment analysis are (1) an increase in applications or software in this field that allows better access to online content and (2) an increase in traffic and content on social platforms where opinions, links, and assessments are shared and general and specialised professional and personal blogs are created. All of them are of interest to different stakeholders, such as companies that offer products and services, their customers and the market in general, suppliers, competition, shareholders, non-profit organisations, governments in their different spheres, from local to international, and society in general. At some point or on a recurring basis, they need to analyse and explore these opinions with the aim of trying to improve their activity and improve their results, not only in the economic field but also in the social and environmental fields. Therefore, in social listening, the contents of an online document, such as a personal or professional blog, a comment or an evaluative review on the website of a hotel or a restaurant or on an online travel platform such as TripAdvisor, are analysed. They analysed the information context as a complete feeling and classified it with the aid of AI as positive, negative or neutral (Altawaiwer and Tiun 2016; Muñoz-Pascual et al. 2021; Saberi and Saad 2017).

Sentiment analysis as a subset of social listening is also known as opinion mining, opinion extraction, sentiment extraction, sentiment classification or sentiment mining (Fang et al. 2018; Keramatfar and Amirkhani 2019). Sentimental analysis tries to classify texts and segregate sentiments or emotions for subjective texts that are mainly related to consumers' opinions or attitudes about products and services. Organisations use this method to obtain information that allows them to understand how customers react to a specific product or service (Sailunaz and Alhajj 2019).

As mentioned above, with the use of Artificial Intelligence (AI) as NPL, text analytics and data mining, identify, extract, and study subjective information. In simpler terms, it classifies a text or feeling as positive or negative. However, sometimes, depending on the general interest in a topic, no feeling appears, and then this situation is classified as neutral.

A bibliographic review (Saberi and Saad 2017; Devika et al. 2016; Szabóová et al. 2020) indicates that sentiment analysis is a complex process that consists of several tasks, such as detecting feelings and analysing their subjectivity, extracting opinions (OM) and trying to guess the orientation of feelings. It is considered a novel and evolving field of research in machine learning (ML), natural language processing (NLP), and computational linguistics. Sentiment analysis is performed at three levels: the word level, sentence level, and document level. The level of analysis determines the task required for the process. The word level is the most complex and the one that provides less satisfactory results due to the difficulty in performing the analysis, while the analysis at the sentence and document level is simpler and offers better results (Balahur et al. 2014).

## Methodology

### Research tasks in sentiment analysis

To contrast the research questions about the use of robots in the tourist environment, we use a social listening method. Social listening is an appropriate method for addressing the objectives of this research since it allows the analysis of content on social media, avoiding any type of bias from the researchers. The use of social media has already been used in other research on human–robot interaction (HRI) (Cramer and Buttner 2011; Zeller et al. 2020).

Sentiment analysis requires you to carry out several research tasks. For example, Szabóová et al. (2020) and De Albornoz et al. (2012) note that they are *subjectivity detection*, which is used in the initial phase of the analysis to separate the objective part from the subjective *part, and polarity classification*, which tries to classify the texts according to three degrees: in positive terms (excellent), negative (poor) or neutral (average). It serves to determine the polarity of the text as a whole, and generally, the polarity change. In this case, text analysis is carried out through complete sentences or parts of them. *Intensity classification* goes beyond polarisation and tries to identify the different degrees of negativity and positivity in a similar way to a Likert scale, e.g., very/strongly negative, negative, fair, positive and very/strongly positive. *Opinion spam* is perhaps one of the biggest existing problems and is a consequence of the increased use of social media.

The fact that there is a greater participation of people in social media is not accompanied by a greater control of the credibility of the opinions that they leave in different places such as a website, a social network, a marketplace, etc. Meanwhile, attempts to verify reviews have grown, and anyone can write what they want, which reduces the credibility and quality of reviews. Szabo et al. (2012) distinguish between three types of opinion spam: the first corresponds to an incorrect opinion, the second corresponds to a review or opinion that is not directly related to the topic, and the last is a distortion when it corresponds to an opinion that has nothing to do with or is not relevant to the opinion analysis. The last is *emotion detection*, which is very similar to *subjectivity detection*. However, in this case, a classification of the existing emotions in a text (anger, joy, sorrow, satisfaction, fear…), their degree of intensity (the degree or quantity of an emotion is analysed, very angry, very happy, a little sad, very happy, etc.), and the detection of the cause of the emotion are carried out to extract the potential causes that involve certain types of emotional expressions that appear in the analysed text (Mohammad and Bravo-Márquez 2018; Gui et al. 2016).

If we analyse the literature (Kawathekar and Kshirsagar 2012; Keramatfar and Amirkhani 2019), the two most commonly used methods to carry out sentiment analysis are the machine learning method and the lexical-based method. However, Devika et al. (2016) point to a third, Rule Based Approach, based on an algorithm that tries to identify subjectivity, polarity or the topic of opinion. It involves a basic routine of natural language processing (NLP) and generally works by creating two lists of words. One of them includes only the positive ones, and the other includes the negative ones. The algorithm goes through the content and finds the words that match the criteria, and after that, the algorithm calculates which type of words is most frequent in the content. If, at the end, there are more positive words, the text is considered to have a positive polarity, and if there are more negatives, the text is considered to have a negative polarity. The rule-based approach can be used for general purposes to determine the tone of messages, which can be useful for customer service. Generally, sentiment analysis focused on the rule-based approach is used as a background for the later use of machine learning.

The machine learning method approach is one of the most popular, and its operation is based on the use of an algorithm that is initially applied to a training dataset before applying it to the real dataset. It is the only approach that truly digs into the content. Unlike the rule-based approach, instead of clearly defined rules, this type of sentiment analysis uses machine learning to discover the essence of the message, which allows it to significantly improve the precision and accuracy of the operation, being able to process information in numerous criteria without becoming too complicated. This approach is the most used in the field of social sciences and management (Keramatfar and Amirkhani 2019). Among the most used methods in this field, Support Vector Machine, Native Bayes Method (Keramatfar and Amirkhani 2019; Thelwall et al. 2012) and N-gram Sentiment Analysis (Devika et al. 2016) are the most common between researchers and practitioners.

The last of the methods used is the lexical-based approach. In this case, lexicon-based techniques work from the assumption that the collective polarity of a text or document is the sum of the polarities of the individual phrases or words that make it up (Mishra et al. 2019; Taboada et al. 2011). It is used more in a linguistic context.

Therefore, according to authors such as Fang et al. (2018), sentiment analysis is suitable for the field of commercial intelligence and in the recommendation environment, as is the case for social media or web marketplaces. It allows a better understanding of the interrelation between the different parties involved in the communication process.

### Data collection

The methodology applied in conducting social media analytics followed four steps (Aral et al. 2013; Fan and Gordon 2014; Stieglitz et al. 2018): discovery, tracking, preparation and analysis. This methodology can be applied in all different domains (Stieglitz et al. 2014). *Discovery* to identify the research domain, in this case social robots. It is important because this topic might not be known a priori and must be discovered first. *Tracking* to select the data source (e.g., Twitter, Facebook), approach, keywords and database structure. *Preparation* to perform data quality analysis and data visualisation. This step is important because it can identify key information and simplify its interpretation. The last step *is analysis* to obtain the relations and conclusions about the data obtained and its adaptation to the domain of research, in our case, hospitality.

In our research (experiment), we analysed Facebook, Instagram and Twitter. However, for privacy reasons, it has only been possible to access the content of tweets on Twitter, where a sample of 8078 tweets has been collected, in which the researchers have not mediated in any way (Ballestar et al. 2020). Analysing these conversations and messages, it is possible to have real-time knowledge in different areas and places that is difficult to appreciate by other means. It is also relevant due to the profitability achieved with its application.

To carry out this experiment, we first monitored the aforementioned social media channels (Facebook, Instagram and Twitter). The aim was to obtain a series of keywords about the use of robots in the tourism sector. Specifically, a library was created with the following keywords in English and Spanish, according to previous research (Qiu et al. 2009). Specifically, in Spanish (Robot Social, Robots sociales, robótica social, interacción humano-robot, sociedad de robots, robots de servicio, humanoide, humanoides, robot antropomorfismo). In English (Social Robot, Social robots, social robotics, human–robot interaction, society of robots, service robots, humanoid, humanoids, and anthropomorphic robot). The experiment was carried out from July 28, 2021, to January 28, 2022 (6 months).

As we mentioned, although three social networks (Twitter, Instagram and Facebook) have been monitored and analysed, data have only been obtained from Twitter, on the one hand, because there are more restrictions on access to public profiles on Instagram and Facebook and because in both, the content about social robots has been zero (Instagram) or very scarce (20) on Facebook. From the results obtained, 64.4% have a neutral result, 20.6% a positive result and the remaining 15% are negative. Finally, the sample obtained is 8078 tweets (7480 from humans and 598 from bots), while 20 messages in Facebook have been dismissed.

Simbiu can analyse all the texts in relation to the keyword that we define, and to analyse the feelings, it relies on the analysis of all the words, comments, hastags and emoticons. The emoticons value them according to their meaning. They do not specify it on the web, but they assess whether the emoticon is positive, negative or neutral (most are ours).

## Results

**Fig. 1:**
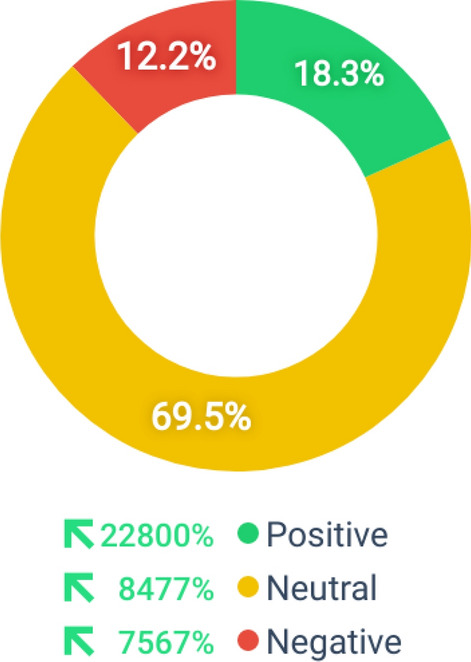
Polarity of sentiment (Sentiment share). *Source*: Own elaboration

The sample by gender obtained is quite balanced, although with a greater male presence, with 60.5% being male and 39.5% being female. The most representative age group was 25–34 years old (49.6%), followed by 18–24 years old (33.1%) and 35–44 years old (12.7%). The main languages in which the information was collected were English (78.4%) and Spanish (9.3%). The sentiment polarity (Fig. 1) is basically neutral (69.5), followed by positive (18.3%) and negative (only 12.2%). Neutral posts are usually informative and are not usually related to a specific type of sentiment (915 negative//1,368 positive//5,195 neutral).

As we have previously mentioned, sentiment analysis in microblogs such as Twitter presents a series of challenges compared to other contexts such as reviews on a web page or in a marketplace. One of the main drawbacks, or challenges, is that microblogs generally contain short messages, informal words, abbreviations, misspellings, and emoticons (Ankit and Saleena 2018; Singh and Kumari 2016). Sentiment analysis on Twitter has been investigated by several authors (Bifet and Frank 2010; Bermingham and Smeaton 2010; Pak and Paroubek 2010, Saif et al. 2014, 2016; Geetha et a. 2017; Sailunaz and Alhajj 2019) since it is a platform that, due to its open source system, allows access to the contents of its users.

**Fig. 2:**
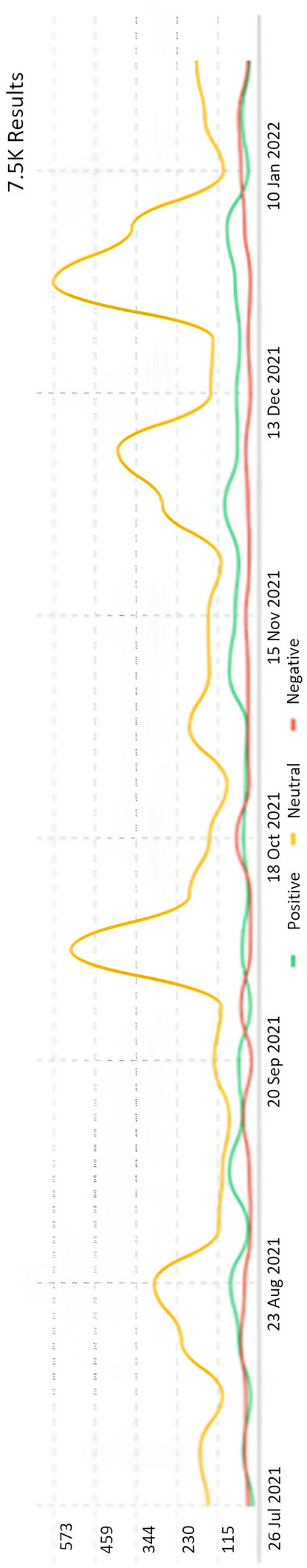
Monitoring over time of the different sentiments. *Source*: Own elaboration

If we monitor the different sentiments over time (Fig. 2), we can see that neutral posts have different intensities by week, while positive and especially negative posts are more regular over time but with a clear lower intensity.

The number of interactions (comments, likes, retweets or shares) with the identified publications was 22,785, and the number of unique users that generated the identified publications was 5967.

**Fig. 3:**
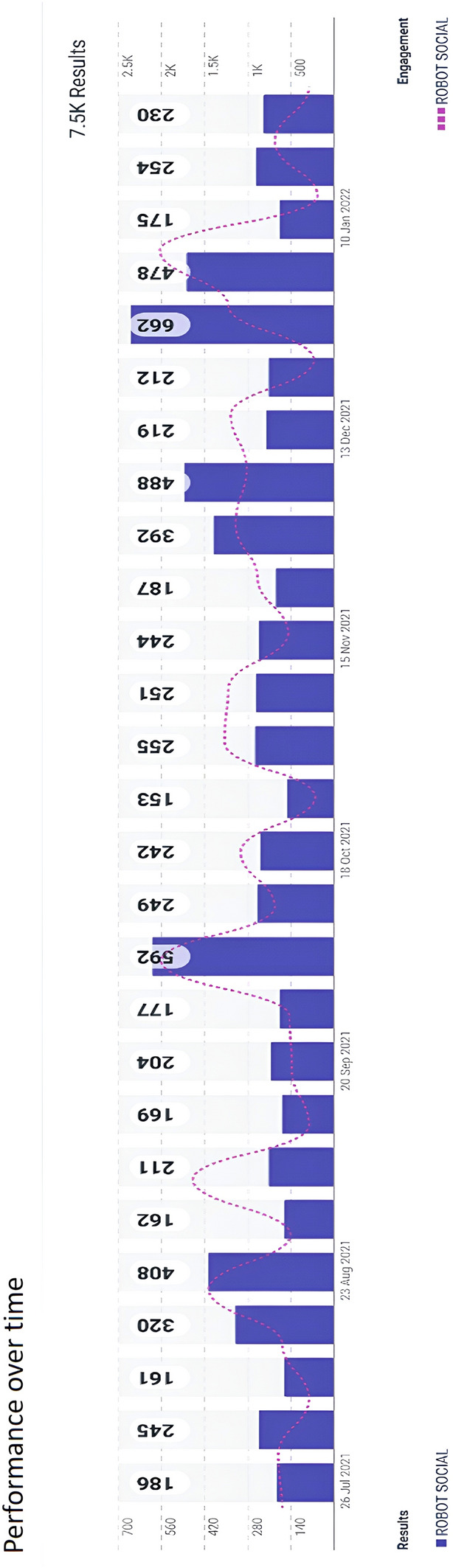
Number of posts and engagement per week. *Source*: Own elaboration

In Fig. 3, we can see in purple the results of the number of posts obtained with the social robot keywords. The pink line (dashed line) represents the engagement of the posts.

As seen, there is not always a correlation between the number of posts and engagement, as there are weeks where fewer posts generate more engagement and weeks where many posts are not always accompanied by the same level of engagement.

Following the research of other authors (Ankit and Saleena 2018; Li et al. 2017; Mishra et al. 2019; Sailunaz and Alhajj 2019; Singh and Kumari 2016), this paper aims to analyse the factors that determine the sentiment (negative, neutral or positive) of the tweets generated on Twitter. For this purpose, we explored different groups of variables: language (English or another language); the number of words in the tweet; tweet content (text, images or videos); tweet generator (bot, external website, Android device or IOS device); the continent where the tweet was generated (Asia, Europe, North America, South America, Australia or Africa); gender; and level of engagement. The following equation shows the relationships analysed, where the dependent variable is sentiment, and the rest of the variables are independent variables:

$$\begin{aligned} Sentiment = & \beta_{0} + \beta_{1} \;English\;language + \beta_{2} \;Word \;Wount + \beta_{3} \;Text + \beta_{4} \;Image \\ & + \;\beta_{5} \;Video + \beta_{6} \;Bot + \;\beta_{7} \;External\;Website + \beta_{8} \;Android + \beta_{9} \;IOS \\ & + \;\beta_{10} \;Asia + \beta_{11} \;Europe + \;\beta_{12} \;NorthvAmerica + \beta_{13} South\;America \\ & + \;\beta_{14} \;Australia + \beta_{15} \;Africa + \beta_{16} \;Gender + \;\beta_{17} \;Engagement + \varepsilon \\ \end{aligned}$$

Table 1 shows the results obtained. The second column of the table shows the variables' effects on sentiment. Regarding the tweet's language, the use of English has a positive and significant impact on sentiment (*β*_1_ = 0.096). The number of words used has a negative and significant effect (*β*_2_ = − 0.003). If the tweet contains images (*β*_4_ = 0.128) or videos (*β*_5_ = 0.158), it generates a significant and positive effect on sentiment, but this effect is not significant if it contains text (*β*_3_ = 0.015).

**Table 1:** Results of hierarchical linnear regression analysis: Regression coefficients (t value). *Source*: Own elaboration

Independent variables ↓	Dependent variable: sentiment	Dependent variable: sentiment (no bot)	Dependent variable: sentiment (bot)	Mean (SD) or % independent var
Constant	− 0.044 (− 1.320)	− 0.026 (− 0.785)	− 0.506 (− 2.836)**	
Language				
English language	0.096 (4.379)***	0.096 (4.378)***	− 0.003 (− 0.023)	90.2%
Number of words				
Word count	− 0.003 (− 4.562)***	− 0.004 (− 6.167)***	0.019 (5.256)***	34.4 (11.5)
Contain				
Text	0.015 (0.687)	0.038 (1.686)	− 0.275 (− 2.606)**	45.2%
Image	0.128 (5.638)***	0.145 (6.308)***	− 0.013 (− 0.113)	36.5%
Video	0.158 (5.186)***	0.166 (5.443)***	0.207 (0.907)	7.0%
Generator				
Bot	− 0.164 (− 6.315)***			7.4%
External website	0.076 (2.697)**	0.072 (2.621)**		5.8%
Android	− 0.055 (− 3.371)***	− 0.055 (− 3.473)***		28.6%
IOS	− 0.028 (− 1.714)	− 0.032 (− 1.970)*		26.6%
Continent				
Asia	0.174 (7.069)***	0.180 (7.322)***	− 0.036 (− 0.241)	7.7%
Europe	0.126 (7.146)***	0.125 (7.058)***	0.017 (0.165)	18.0%
North America	0.050 (3.124)**	0.049 (3.081)**	0.023 (0.211)	22.9%
South America	0.034 (0.866)	0.028 (0.694)	0.250 (1.398)	2.7%
Australia	0.130 (2.348)*	0.128 (2.347)*	0.044 (0.064)	1.3%
Africa	0.001 (0.016)	− 0.005 (− 0.088)	− 0.123 (− 0.431)	1.2%
Gender				
Female	0.001 (0.092)	− 0.003 (− 0.207)	0.052 (0.545)	26.9%
Engagement	0.090 (4.581)***	0.091 (4.712)***	0.116 (0.705)	2.82 (31.6)
VIF( ≤)	3.210	3.203	3.261	
*R*^2^	0.045	0.042	0.081	
*F*	22.219	20.273	3.956	
Sig. *F*	0.000	0.000	0.000	

*VIF* Variance inflation factor (VIF above 4 indicates that multicollinearity might exist). *R*^2^: R-squared is a statistical measure that represents the proportion of the variance explained. *F*: F value, if the significance associated with the F value is lower than 0.05, then the independent variables reliably predict the dependent variable. *p < 0.05; **p < 0.01; ***p < 0.001

Concerning the tweet generator, if it comes from a Bot (*β*_6_ = − 0.164) or an Android device (*β*_8_ = − 0.055), the effect on sentiment is negative and significant. If the generator is an External Website, the effect is significant and positive (*β*_7_ = 0.076). Tweets generated with an IOS device have a negative but not significant effect (*β*_9_ = − 0.028, nonsignificant).

Regarding the continent of origin of the message, tweets from Asia (*β*_10_ = 0.174), Europe (*β*_11_ = 0.126), North America (*β*_12_ = 0.050) and Australia (*β*_14_ = 0.130) have a significant and positive effect on sentiment. However, tweets from South America (*β*_13_ = 0.034) and Africa (*β*_15_ = 0.001) have no significant impact on sentiment.

Unlike other studies in the field of new technologies and emotions (Garaus and Wolfsteiner 2023), gender does not influence sentiment towards robots. In our study, gender does not significantly affect sentiment (*β*_16_ = 0.001). Finally, engagement positively and significantly impacts sentiment (*β*_17_ = 0.090).

We consider it interesting to compare the results considering the influence of bots. Differences exist in whether the tweet generator was a bot when analysing sentiment antecedents (Columns 3 and 4 of Table 1). When the origin of the tweets was not a bot, the results are similar to the general pattern. However, in this scenario, the effect of using an IOS device on sentiment is significant and negative (-0.028). When the tweet source is a bot, the sentiment antecedents are only the number of words (0.019) and that the tweet includes text (-0.275); in the first case, the effect is positive, and in the second case, it is harmful. A message generated by a bot is associated with negative sentiment, tempered by the number of words in the tweet.

**Fig. 4:**
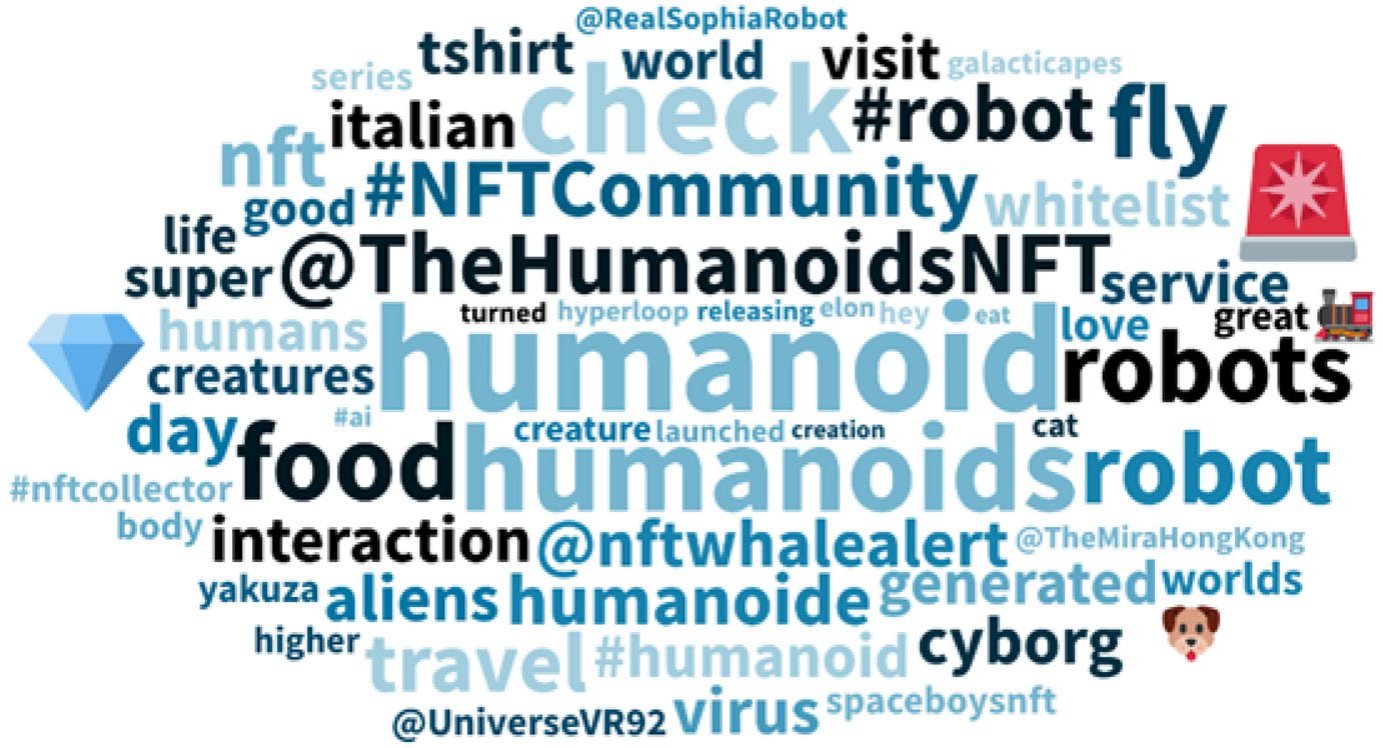
Word clouds of trends. *Source*: Own elaboration

**Fig. 5:**
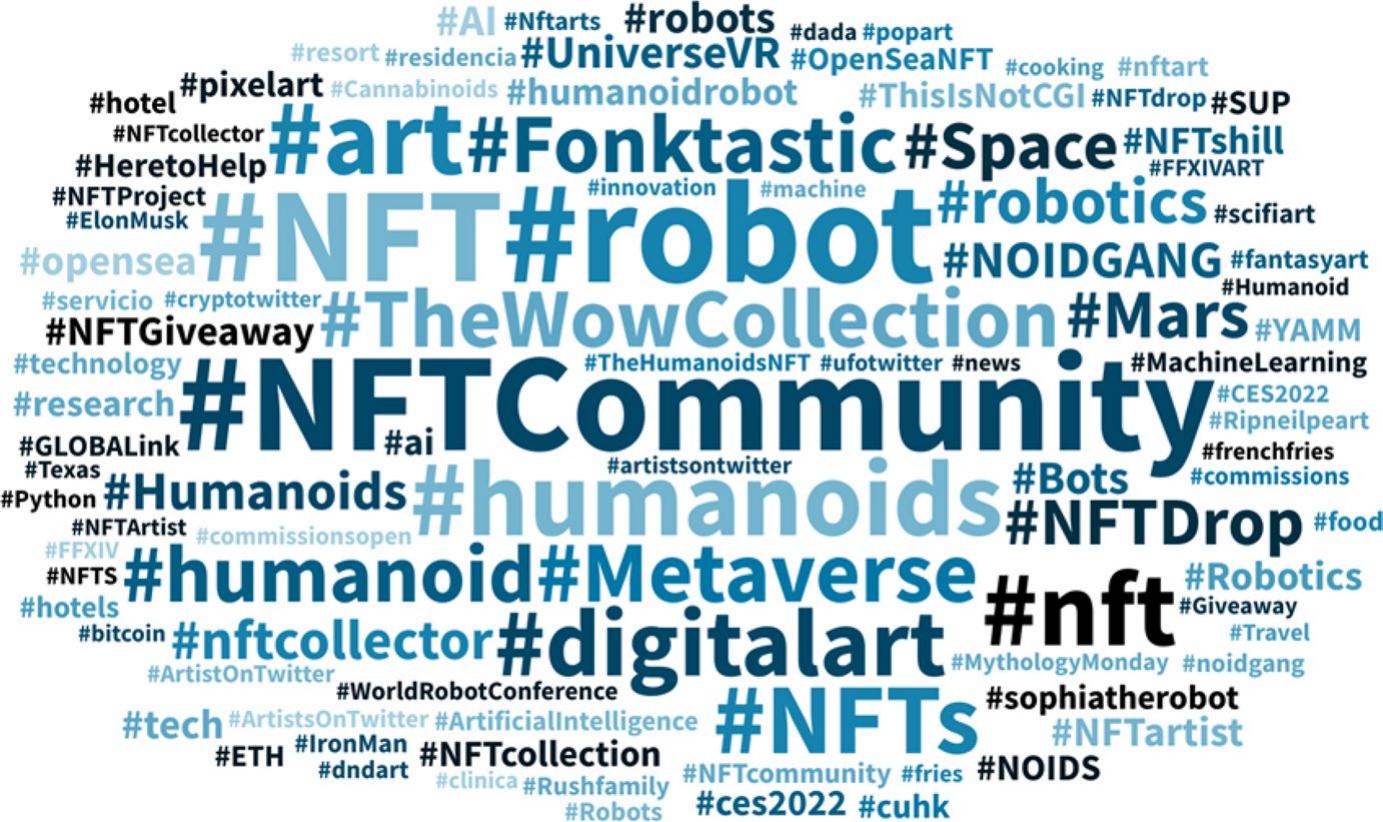
Word clouds of hashtags. *Source*: Own elaboration

Within the exploratory data analysis, we basically analysed the most common words that appear in the content on Twitter. For them, we use word clouds because they offer a better visual representation and help to make comparisons between trends and hashtags. The higher the usage frequency of a word, the larger its presence in the word cloud (Geetha et al. 2017). Figures 4 and 5 show that certain terms have been used more frequently. In the case of trends, the most frequently used term was "humanoid", with a frequency of 65.3%, and the second most frequently used term was "humanoids", with a frequency of 15.2%. In hashtags, the most frequently used term is "#humanoids", with a frequency of 25.3%, followed by "#NFTCommunity", with a frequency of 18.3%.

In Fig. 4, we can see the word clouds of trends, where “humanoid”, “check”, “food” and “robots” are the most used words. Perhaps the most notorious cases of using robots to serve food or drinks in restaurants or catering have an influence on the content of social media. In this case, different emoticons appear, for example, a diamond, which can be understood as something sophisticated and innovative. Another emoticon is a police siren, calling attention to something, for the novelty effect and its use, social or innovation consequences.

If we analyse the word clouds of hashtags (Fig. 5), in this case, #NFT, with different acceptations (#NFT, #NFTs, #NFTCommunity, #NFT arts, #nft), is the most relevant. However, it appears #robot, #humanoids, which have a strong relationship within our research.

**Fig. 6:**
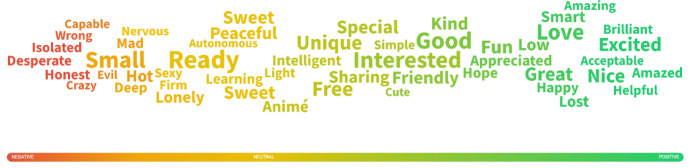
Sentiment controllers. *Source*: Own elaboration

Finally, in Fig. 6 we can see the most representative words that the software identifies to assess the sentiments. Although they are not all there, they make this image with the most representative ones. (Sentiment controllers). On the left, the words that identify more with negative sentiments appear (“Desperate, Wrong, Honest and Crazy” are the most usual), while on the right appear those that identify with positive ones (“Nice, love, excited and great “), and in the middle, those that identify with the most neutral sentiments (“Good, Interested, Unique, Special, Free”). In this case, previous HRI experiments may explain this situation.

## Discussion and conclusion

### Discussion

The advancement of innovations and technology and constant changes in our social and health environment are increasingly present in people's daily lives (Åström et al. 2022; Kraus et al. 2019). In this new context, innovations based on social robots are appearing strongly. In the tourism sector, their presence, in addition to being an innovative way of seeking interaction with the client, can help alleviate some of the problems that have arisen as a result of COVID-19 (Breier et al. 2021). This can help tourism companies, and especially hotel companies, recover from the economic crisis, social and health crisis in which they are immersed (Pillai et al. 2021; Hidalgo et al. 2022). In this research, we proposed to analyse, as a preliminary step in the near future, the opinion that the use of social robots in the tourism sector, especially in the hotel sector, arouses. For this, we have relied on Simbiu, a software tool that follows the operating basis of Talkwalker. Efforts to improve these relationships through the interpretation of social behaviours are becoming more frequent in research on social robotics.

In the creation of the library of words (keywords), English and Spanish were used, and the results obtained indicate that the use of English has a positive impact on sentiment, and these results are in harmony with the results of other investigations (Bhardwaj and Kumar 2016; Arun and Srinagesh 2020) where sentiment analysis using the English language tends to give better results than other languages. Another result to highlight is that the number of words used has a negative and significant effect. The use of images or videos generates a more positive effect on sentiment than if the tweet has only text. This result is very similar to other previous research (Yost et al. 2020; Zeller et al. 2020), where the use of images and videos is generally better accepted in messages than in those messages that only show text and tend to generate more engagement. This circumstance could also explain the fact that the number of words significantly and negatively influences the sentiment of the tweets, perhaps because it is expected to be accompanied by some type of image, video, external link or emoticon.

After all these comments, we believe that it is necessary to ask ourselves a series of questions. For example, regarding languages, and given that English and Spanish are the two majority languages in our sample, do Anglophones perceive social robots better than Latinos? Regarding the operating system from which the comments come, does the device influence the perception of social robots, that is, are there differences between IOS and Android users? Regarding the contents of the messages, are there significant differences between the profiles of people who post videos and photos versus those who use only text?

The origin of most of the analysed tweets comes from Asia, Europe, North America, and Australia, and they have a positive effect on sentiment. The fact that these are the geographical areas where many of the most developed countries are concentrated means that they are also the places where these robots are used most frequently and therefore where there may be greater social acceptance (de Kervenoael et al. 2019; Mishra et al. 2019; Pillai et al. 2021).

In other words, gender does not significantly affect sentiment, but engagement positively and significantly impacts sentiment, perhaps because a person who identifies with the use of robots or with the use of new technologies will generally show a positive predisposition towards their interaction and use.

Another aspect that we must highlight is the tweet generator because in the literature, there is a real controversy about the acceptance of the use of bots in sentiment analysis (Dickerson et al. 2014; Rodríguez-Ruíz et al. 2020). In our case, if we consider the use of bots, if it comes from a Bot or an Android device, the effect on sentiment is negative and significant. However, if the generator is an external website, the effect is significant and positive, and tweets generated with an IOS device have a negative but not significant effect. In this case, we believe it is necessary more research to corroborate these results and to understand more in this scope.

To know more about the use or absence of bots in Twitter, we differentiate whether the tweet generator was a bot or not to analyse sentiment antecedents (Columns 3 and 4 of Table 1). When the origin of the tweets was not a bot, the results are similar to the general pattern. However, when the tweet source is a bot, the sentiment antecedents are only the number of words (0.019) and that the tweet includes text (-0.275); in the first case, the effect is positive, and in the second case, it is harmful. A message generated by a bot is associated with negative sentiment, tempered by the number of words in the tweet. These results are very similar to those obtained by Dickerson et al. (2014), where humans tend to express stronger positive sentiment and negative sentiment (but slightly more nuanced) than bots, and in general, humans disagree more with the general sentiment of the application's Twitter population than bots.

Finally, our results offer that, with the use of social listening, we can obtain more information about the acceptance of social robots in the tourism sector and specifically in hospitality. The final consideration is that positive image is more significant than negative, but neutral opinions are the most important in the social media analysed. This is very important because the use of social robots in the hospitality sector can help to improve customer experience and push employees' work towards more relevant tasks, leaving the more tedious or dangerous ones (cleaning in hard-to-reach areas, handling hot products, etc.) to social robots (Rojas-Cordova et al. 2022). It would favour the integration of these robots into the daily tasks performed by employees without posing a threat to their jobs, taking into consideration their usefulness and ease of use from the perspective of both employees and users.

### Theoretical contributions

Our main contribution with this paper is the use of a big data technique that allows us to identify the polarity of sentiment towards a technological innovation applied to a hospitality context, such as social robots. This introduces a new line of research based on the analysis of unstructured text published on social networks using AI algorithms based on machine learning. Compared to the traditional use of surveys, big data provides greater representativeness and greater spontaneity, although it also presents problems of missing values and unstructured data.

Although service robots are being deployed at a rapid rate, the sentiment generated by this disruptive technology is still neutral. The social acceptance of a technological innovation is a key aspect for its implementation. Social listening is a methodology for monitoring the evolution of the social acceptance of a technology. Although social media has limitations in terms of measuring variables with precision, the use of proxy variables such as user engagement (number of retweets, number of likes) and the adaptation of the message to customer features can accelerate the social acceptance of a disruptive technology such as social robots.

### Practical implications

Concerning the tourism sector, we highlight the following implications for management. Those responsible for tourist organisations or destinations should encourage the proper use of words to describe tourists' experiences. Excessive use of words in the tweets potentiated the negative sentiment, and these managers must promote images and videos in tweets to positively affect sentiment. The presence of social robots can help, from an innovation standpoint, to collect information on what customers need beyond interaction with hotel employees (reception staff, cafeterias, cleaning staff, etc.), in a way that, by not interacting with people, they can disinhibit themselves and behave and express themselves in a more casual, accessible way. However, for this, it is essential that robots can respond and interact with people naturally and intuitively (Breazeal 2002; Danner‑Schröder 2021), and it is important that social robots provide practical benefits both to clients or hotel guests and to the hotel itself in general (Tulli et al. 2019). However, the use of innovations such as social robots is not indifferent to people or society in general. Its use draws attention, generates differentiation using innovations of a technological nature, and generates a media environment that helps spread the image of the hotel in the physical environment (wom) and in the digital environment (ewom), enhancing its brand recognition.

Tweets from an external website that contain videos or images from that website generate positive sentiment; therefore, it is convenient to encourage tourists to create tweets that contain photographs and videos from the websites of the accommodation or tourist destinations to help improve awareness of the hotel, its brand image and improve its positioning as an innovative company.

### Limitations and further research

Our main limitations are found in the level of knowledge of the tools used, and we must continue training to improve this aspect. We need to improve the refined data collection process to obtain more explanatory variables and obtain more compelling information. Simbiu is a software tool that is constantly improving, which forces us to routinely focus on and improve our level of knowledge. The size of the sample 8078 tweets, although important, is not very high, and the period of time in which the follow-up and analysis has been carried out (6 months), given the novelty and specificity of the chosen topic, make it necessary to expand the sample and the space of time in future research to obtain data that would allow obtaining more conclusive results or reaffirm those obtained in this paper. Additionally, the problem of not being able to obtain more information from other social networks such as Instagram or Facebook should be addressed in future research. Other social networks such as YouTube or Vimeo should also be incorporated. We hope that in new updated versions of Simbiu, this situation can be resolved and access to these or other social networks is allowed. The changing nature of the COVID-19 situation makes it challenging to keep abreast of the latest results. The level of vaccination and how it evolves by country, its economic, social and health consequences, influences people based on their own experiences Finally, as a consequence of our results, social listening and sentiment analysis should continue to be investigated through new fieldwork on the polarity of sentiment in HRI, both in the context of hospitality and in others such as catering, gastronomy, or in other domains such as retail or health (hospitals, geriatrics, …).

## Data Availability

The datasets generated and analysed during the current study are not publicly available because they constitute an excerpt of research in progress but are available from the corresponding author on reasonable request.
